# Comparison of the short-term efficacy of different Amplatzer models and similar occluders in the treatment of patent foramen ovale

**DOI:** 10.3389/fcvm.2023.1092465

**Published:** 2023-07-11

**Authors:** Yuxuan Lou, Yang Hua, Jing Shi, Fengze Yang, Yifei Wang, Yang Yang, Wei Sun, Xiangqing Kong, Hao Zhang

**Affiliations:** ^1^ Department of Cardiology, The First Affiliated Hospital of Nanjing Medical University, Nanjing, China; ^2^ Southeast University School of Medicine, Nanjing, China

**Keywords:** patent foramen ovale, cryptogenic stroke, migraine, closure, Amplatzer occluder

## Abstract

**Objective:**

To compare the recent efficacy and safety of different Amplatzer models and similar occluder in the treatment of patent foramen ovale (PFO).

**Methods:**

Patients with PFO complicated with cryptogenic stroke or migraine who underwent transcatheter closure of PFO in the First Affiliated Hospital of Nanjing Medical University from September 2019 to March 2021 were selected. Patients were grouped according to the type of occluder device. The basic data of the patients were collected and followed up within 1 year after occlusion. Effectiveness was defined as no recurrence of stroke/remission of migraine symptoms and a negative postoperative foaming test, and safety events were counted as the combined results of serious adverse events.

**Results:**

A total of 92 patients were selected, including 45 cases in the symmetrical group and 47 cases in the asymmetric group. There were no serious adverse events in the 2 groups during follow-up. 3 days and 1 month after occlusion, the number of shunt patients in the asymmetric group was significantly less than that in the symmetric group (*χ*^2^ = 5.484, *P* = 0.019; *χ*^2^ = 5.146, *P* = 0.023). The negative rate of blocked residual shunts in the asymmetric group was higher than that in the symmetric group at 1, 3, 6 and 12 months after occlusion (*χ*^2^ = 6.473, *P* = 0.011; *χ*^2^ = 4.305, *P* = 0.038; *χ*^2^ = 4.842, *P* = 0.027; *χ*^2^ = 4.034, *P* = 0.045). Headache in migraine patients in the asymmetric group was significantly better than headache in patients in the symmetric group (*P* = 0.038; *P* = 0.049).

**Conclusion:**

Asymmetric Amplatzer and similar occluders provide greater efficacy in short-term occlusion than symmetric ones.

## 1. Introduction

The foramen ovale is an open structure in the middle of the atrial septum that is covered by a thin slice of the primary septum. During embryonic development, the foramen ovale serves as an important conduit for oxygen and nutrients from maternal umbilical vein blood to the fetal left cardiac system. After birth, the primary septum and secondary septum of most newborns will gradually adhere and fuse to form a permanent atrial septum. Patent foramen ovale (PFO) is described when the foramen ovale has remained open past the age of 3. Approximately a quarter of the adults in the population have this condition. Statistics from Western countries show that the incidence of PFO is approximately 34.3% in adults under the age of 30 and 20.2% in the elderly aged 80–99 (1). In the past, it was believed that PFO did not cause any serious clinical problems, as the shunt flow of PFO is typically very small under normal circumstances. However, with increasing publication of clinical research results, it has become known that there is an association between PFO mediated left-right shunting and cryptogenic stroke (CS) (2) and migraine (3). Transcatheter closure of PFO can effectively prevent the recurrence of CS and migraine.

At present, China only approves Amplatzer PFO occluders or similar domestic occluders for clinical use (4). There are two main types of PFO occluders in China: one has a completely symmetrical (24/24 mm; 28/28 mm; 30/30 mm) of the left and right discs, and the other has an asymmetric (18/24 mm; 18/25 mm; 22/28 mm; 25/35 mm) with the diameter of the right disc slightly larger than that of the left disc and slightly concave. At present, there is no relevant study on the difference in effectiveness and safety between symmetric and asymmetric Amplatzer and Amplatzer-like occluders. This study aims to evaluate whether PFO patients with CS or migraine who have indications for interventional therapy can benefit from occlusion treatment and to compare the short-term effectiveness and safety of the two main types of occluders, thereby providing a basis for improving the level of PFO treatment in the clinic.

## 2. Material and methods

### 2.1. Objects

This study was a single-center, retrospective clinical study. Patients with PFO complicated with CS or migraine who received treatment by transcatheter closure of PFO in the First Affiliated Hospital of Nanjing Medical University from September 2019 to March 2021 were selected. Inclusion criteria: (1) Contrast transcranial Doppler (c-TCD) screening was grade 3; (2) PFO with stroke/transient ischemic attack (TIA) or history of migraine not easily controlled by drugs; (3) patients who received transcatheter closure of PFO and regular follow-up after occlusion. Exclusion criteria: (1) stroke etiology determined after investigation (excluded based on TOAST classification (5); (2) definite secondary headache; (3) age <16 years old or >70 years old; (4) other organic heart diseases requiring surgical intervention; (5) abnormal coagulation mechanism, autoimmune diseases, serious heart, liver, kidney failure, etc., who are unable to complete transthoracic echocardiography (TTE), transesophageal echocardiography (TEE), c-TCD examination and interventional occlusion; (6) unwillingness to receive closure treatment and follow-up. The general information of patients and relevant examination results, including sex, age, height, weight, blood pressure, heart rate, and results of TTE, TEE, and c-TCD, were recorded. The study design was approved by the Ethics Committee of the First Affiliated Hospital of Nanjing Medical University.

### 2.2. Instruments and occlusion procedure

#### 2.2.1. C-TCD

MVU-6300 Doppler ultrasound diagnostic instrument with a probe frequency of 2 MHz was applied to detect the change in TCD. The right-to-left shunt (RLS) can be classified as follows according to the 2017 Chinese neuroultrasound operation specification (6).

**Table:** 

Grade	Number of unilateral MB	Number of bilateral MB	
Grade 0 (none, negative)	0 MBs	0 MBs	Calculated at higher score
Grade 1 (mild RLS)	1–10 MBs	1–20 MBs	
Grade 2 (moderate RLS)	>10 MBs, but not into a curtain	>20 MBs, but not into a curtain	
Grade 3 (large RLS)	Curtain or shower type that does not differentiate a single MB signal		

MB, microbubble.

#### 2.2.2. TTE and TEE

Philips-ie33 color Doppler ultrasound diagnostic instrument was used to comprehensively evaluate the size, structure and function of the heart with a probe frequency of 2–4 MHz.

A Philips-epiq7c ultrasonic diagnostic instrument was used to observe whether there was a shunt at the level of the atrial septum.

#### 2.2.3. Transcatheter closure therapy

Patients who met the indications in the guidelines for percutaneous interventional therapy of common congenital heart diseases (7) were treated according to the operating standards. All transcatheter closure were performed by the same operator.

Migraine disability assessment (MIDAS) questionnaire score: According to the principle of the double-blind method to evaluate migraine level in patients, the degree of disability can be assigned to one of four levels according to points: 0–5 points is grade I, and the degree of disability is mild; 6–10 points is grade II, mild disability; 11–20 points is grade III, moderate degree of disability; >21 is grade IV, severe disability.

### 2.3. Grouping method

According to the occluder selected for interventional occlusion, the selected patients were divided into a symmetrical group (occluder size: 24/24 mm; 28/28 mm; 30/30 mm) and an asymmetric group (occluder size: 18/24 mm; 18/25 mm; 22/28 mm; 25/35 mm). The choice of occluder model was determined by the PFO separation width, tunnel length, and shape, as well as ultrasound findings. According to current national and international guidelines, a moderate-sized 18/25 mm occluder is typically selected as the first choice for most PFO. However, for complex PFO, such as those with atrial septal aneurysm (ASA), long tubular PFO, or secondary septal hypertrophy, there is concern that the occluder disc may abrade the aorta, in which case a 25/35 mm or 30/30 mm PFO occluder may be directly chosen.

### 2.4. Follow-up

The patients were subjected to a comprehensive follow-up schedule that included visits at 3 days, 1 month, 3 months, 6 months, and 12 months post-occlusion. TTE, c-TCD, dynamic electrocardiogram and other examinations were to be performed in each follow-up to determine whether there was pericardial effusion or tamponade, occluder embolization or displacement, atrial fibrillation and other related complications. Magnetic resonance imaging (MRI) was performed at 6 months and 12 months after the occlusion, in migraine patients, the MIDAS test was repeated to evaluate the efficacy of occlusion. The second recheck was performed to confirm complete blockage of PFO without residual RLS. Effectiveness was defined as no recurrence of stroke/remission of migraine symptoms and a negative foaming test after occlusion. Safety events were noted as the comprehensive results of serious adverse events related to the occlusion of occlusion or devices (such as pericardial effusion or tamponade, occlusion or displacement of occluder, arrhythmia, etc.).

### 2.5. Statistical analysis

SPSS 24.0 software was used to process the data. The counting data are expressed as the number and percentage of cases, and the results of continuous variables with a normal distribution of measurement data are expressed as the mean ± standard deviation. Independent sample *t* tests were used for intergroup comparisons. The data of categorical variables were compared by the chi square test. When the expected frequency of each cell was ≥5 and the total number of cases was ≥40, the Pearson chi square test was used. If the expected frequency of any cell was ≥1 but <5, the continuity correction chi square test was used. When the expected frequency of each cell is less than 1, or the total number of cases is less than 40, Fisher exact probability method is used. *P* < 0.05 was considered statistically significant.

## 3. Results

### 3.1. Basic information of patients

The type of occluder was chosen based on the admission time, with symmetric occluders applied for patients admitted from September 2019 to June 2020 and asymmetric occluders for those admitted from July 2020 to March 2021. By March 2021, 92 patients had been enrolled, including 45 patients in the symmetrical group and 47 patients in the asymmetric group. Among the 92 patients, 24 were male and 68 were female, and ages ranged from 16 to 70 (41.70 ± 13.67) years. There were 53 patients of migraine (57.61%) and 39 patients of cryptogenic stroke (42.39%). Two sets of baseline characteristics are described in Table 1.

**Table 1:** Patient characteristics.

Variables	Symmetry group (*n* = 45)	Asymmetric group (*n* = 47)	Test statistics	*P-*value
Age (years)	40.01 ± 12.73	41.29 ± 15.22	*t *= −0.428	0.670
Female (%)	35 (77.78)	33 (70.21)	*χ*^2^ = 0.682	0.409
Systolic (mmHg)	122.28 ± 13.28	120.56 ± 15.29	*t *= 0.535	0.594
Diastolic (mmHg)	79.97 ± 8.41	77.51 ± 10.23	*t *= 1.173	0.244
Heart rate (times/min)	79.11 ± 8.97	77.02 ± 9.00	*t *= 10.29	0.307
Cryptogenic stroke (%)	20 (44.44%)	19 (40.43%)	*χ*^2^ = 0.152	0.696
Migraine (%)	25 (55.56%)	28 (59.57%)	*χ*^2^ = 0.152	0.696
Hypertension (%)	5 (11.11%)	8 (17.39%)	*χ*^2^ = 0.662	0.415

The transthoracic echocardiography results of the two groups were as follows (Table 2): There was no significant difference in aortic root diameter, left atrial diameter, left ventricular diastolic diameter, left ventricular systolic diameter, ventricular septal thickness, left ventricular posterior wall thickness, right atrial diameter, right ventricular diastolic diameter, left ventricular shortening fraction or left ventricular ejection fraction between the symmetrical group and the asymmetric group (*P *> 0.05). There were 21 cases (46.67%) of atrial septal aneurysm in the symmetrical group and 18 cases (38.30%) in the asymmetrical group. There was no significant difference in the results (*P *> 0.05).

**Table 2:** Transthoracic echocardiography.

Variables	Symmetry group (*n* = 45)	Asymmetric group (*n* = 47)	Test statistics	*P-*value
Aod (mm)	29.22 ± 2.90	28.88 ± 3.07	*t *= 0.499	0.619
LAD (mm)	31.35 ± 3.48	31.20 ± 3.74	*t *= 0.191	0.849
LVDd (mm)	45.78 ± 3.49	46.05 ± 3.36	*t *= −0.341	0.734
LVDs (mm)	29.86 ± 2.37	30.05 ± 2.21	*t *= −0.353	0.725
IVS (mm)	9.70 ± 0.57	9.55 ± 0.71	*t *= 1.079	0.284
LVPW (mm)	9.70 ± 0.57	9.48 ± 0.77	*t *= 1.493	0.140
RAD (mm)	30.11 ± 3.18	30.19 ± 3.56	*t *= −0.109	0.914
RVDd (mm)	29.92 ± 3.58	30.33 ± 3.00	*t *= −0.552	0.582
FS (%)	34.74 ± 1.45	34.71 ± 1.97	*t *= 0.076	0.940
EF (%)	64.07 ± 2.11	63.91 ± 2.53	*t *= 0.299	0.765

Aod, inner diameter of the ascending aorta; LAD, left atrial diameter; LVDd, left ventricular diastolic inner diameter; LVDs, left ventricular systolic inner diameter; IVS, interventricular septal thickness; LVPW, posterior wall thickness of the left ventricle; RAD, right atrial diameter; LVDd, right ventricular diastolic inner diameter; FS, fractional shortening; EF, ejection fraction.

Table 3 shows the results of PFO morphology under transesophageal echocardiography in both groups, with no significant differences in PFO separation width, tunnel length, ASA, proportion of secondary septal hypertrophy, or complex PFO between symmetric and asymmetric patients (*P *> 0.05). Occluder model selection was made based on ultrasound results, with 25/35 mm or 30/30 mm occluders selected for complex PFO (4).

**Table 3:** PFO morphology.

Variables	Symmetry group (*n* = 45)	Asymmetric group (*n* = 47)	Test statistics	*P-*value
PFO separation width (mm)	3.21 ± 0.80	3.01 ± 0.70	*t* = 1.239	0.219
PFO tunnel length (mm)	6.34 ± 1.27	6.19 ± 1.01	*t* = 0.627	0.532
ASA (%)	14 (31.11%)	13 (27.66%)	*χ*^2^ = 0.132	0.716
Secondary septal hypertrophy (%)	4 (8.89%)	2 (4.26%)	*χ*^2^ = 0.810	0.368
Complex PFO (%)	21 (46.67%)	18 (38.30%)	*χ*^2^ = 0.659	0.417

ASA, atrial septal aneurysm.

In the symmetrical group, 13 cases (28.89%) were 24/24 mm, 11 cases (24.44%) were 28/28 mm, and 21 cases (46.67%) were 30/30 mm. In the asymmetric group, occluder devices were selected as follows: 18/24 mm in 5 cases (10.64%), 18/25 mm in 19 cases (48.94%), 22/28 mm in 5 cases (10.64%), and 25/35 mm in 18 cases (38.30%). There was no significant difference in the diameter of the right disc between the symmetrical group and the asymmetrical group [(27.78 ± 2.57 mm) vs. (29.07 ± 4.90 mm), *P* = 0.121], and there was a significant difference in the diameter of the left disc [(27.78 ± 2.57 mm) vs. (21.09 ± 3.36 mm), *P *< 0.001].

### 3.2. Results of transcatheter closure

There was no significant difference in the operation x-ray exposure time or hospitalization time between the symmetrical group and the asymmetric group (exposure times 3.51 ± 1.70 min and 3.48 ± 3.13 min, respectively, *P* = 0.966; and hospitalization times 4.02 ± 0.54 d and 4.07 ± 0.44 d, respectively, *P* = 0.680). No occlusive complications, such as pericardial effusion or tamponade, occluder embolism or displacement, during or 24 h after occlusion in the symmetrical group or the asymmetric group. No new serious arrhythmia was found on the dynamic electrocardiogram after occlusion. Reexamination with c-TCD within 3 days after the occlusion showed that there were still large RLS (in 21 patients from the symmetrical group and 11 patients from the asymmetric group), statistical analysis showed that the difference between the two groups was statistically significant (*χ*^2^ = 5.484, *P* < 0.05) (Supplementary Table S1); there were 11 patients who were negative for RLS in the symmetric group and 20 such patients in the asymmetric group, statistical analysis showed that there was no significant difference between the two groups (*χ*^2^ = 1.056, *P* > 0.05) (Table 4).

**Table 4:** Comparison of negative RLS after occlusion between the symmetric group and asymmetric group.

Item	Symmetry group	Asymmetric group	*χ* ^2^	*P-*value
Within 3 days after occlusion	*n* = 45	*n* = 47	1.056	0.304
Negative RLS	11	20		
Positive RLS	24	27		
1 month after occlusion	*n* = 42	*n* = 46	6.473	0.011
Negative RLS	16	30		
Positive RLS	26	16		
3 months after occlusion	*n* = 37	*n* = 45	4.305	0.038
Negative RLS	18	32		
Positive RLS	19	13		
6 months after occlusion	*n* = 35	*n* = 41	4.842	0.027
Negative RLS	17	30		
Positive RLS	18	11		
12 months after occlusion	*n* = 35	*n* = 39	4.034	0.045
Negative RLS	25	35		
Positive RLS	10	4		

### 3.3. Follow-up

The patients were followed up until March 2022, with a median follow-up of 12 months. The occluder position of all patients was good, and there were no complications, such as occluder displacement, hematogenous infection, thrombosis, and pericardial effusion.

One month after occlusion, 88 patients underwent c-TCD reexamination in the outpatient department, and 4 patients were lost to follow-up. Fourteen patients in the symmetrical group still had large RLS, and 6 patients in the asymmetric group still had large RLS. Statistical analysis showed that the difference between the two groups was statistically significant (*χ*^2^ = 5.146, *P* < 0.05) (Supplementary Table S1). There were 16 patients who were negative for RLS in the symmetric group and 30 such patients in the asymmetric group. Statistical analysis showed that the difference between the two groups was statistically significant (*χ*^2^ = 6.473, *P* < 0.05) (Table 4).

Three months after occlusion, 82 patients completed c-TCD follow-up, and 6 patients were lost to follow-up. Three patients in the symmetrical group still had large RLS, and no patient in the asymmetrical group had large RLS (*P* > 0.05) (Supplementary Table S1). There were 18 patients who were negative for RLS in the symmetric group and 32 such in the asymmetric group. Statistical analysis showed that the difference between the two groups was statistically significant (*χ*^2^ = 4.305, *P* < 0.05) (Table 4).

Six months after occlusion, 76 patients completed c-TCD follow-up, 6 patients were lost to follow-up. There were 17 patients who were negative for RLS in the symmetric group and 30 such patients in the asymmetric group. Statistical analysis showed that the difference between the two groups was statistically significant (*χ*^2^ = 4.842, *P* < 0.05) (Table 4). At 12 months after occlusion, 74 patients completed c-TCD follow-up, 2 patients were lost to follow-up. There were 25 patients who were negative for RLS in the symmetric group and 35 such patients in the asymmetric group, the remaining 14 patients had moderate/mild shunts. Statistical analysis showed that the difference between the two groups was statistically significant (*χ*^2^ = 4.034, *P* < 0.05) (Table 4).

During the follow-up period, no safety events occurred in either group, and there was no symptom recurrence in either group. There was no significant difference in the MIDAS scores between the symmetric group and the asymmetric group before occlusion (*P* = 0.528) (Table 5). The MIDAS scores of the symmetric group were significantly higher than those of the asymmetric group during 6 and 12 months follow-up after occlusion (*P* = 0.038; *P* = 0.049) (Table 5).

**Table 5:** Comparison of scores of migraine patients.

Score	Symmetry group (*n* = 25)	Asymmetric group (*n* = 28)	*t*-value	*P*-value
MIDAS score before closure	42.04 ± 14.13	39.00 ± 19.82	0.636	0.528
MIDAS score 6 months after closure	20.56 ± 12.95	14.36 ± 7.97	2.125	0.038
MIDAS score 12 months after closure	19.24 ± 11.24	14.00 ± 7.45	2.021	0.049

## 4. Discussion

Studies have shown that CS or migraine with anatomic/clinical risk factors are related to PFO. Transcatheter closure of PFO can effectively reduce the recurrence of CS or migraine. Several studies published in 2017, including the CLOSE (8), REDUCE (9), and RESPECT (10) studies, as well as the DEFENSE (11) study on high-risk PFO in Asia and the five-year follow-up of the REDUCE (12) study in 2021, have demonstrated that transcatheter closure of PFO can decrease the risk of stroke recurrence compared to drug treatment alone. The secondary endpoints of large randomized controlled clinical trials such as the PRIMA study (13) and PREMIUM study (14, 15) related to the treatment of migraine by PFO occlusion also suggested that PFO interventional occlusion can improve the migraine symptoms of patients. With the accumulation of evidence-based medical information, relevant guidelines and expert consensus have taken transcatheter closure of PFO and blocking of right-to-left shunt (especially large RLS) as routine treatments for migraine and cryptogenic stroke, including primary prevention and prevention of recurrence. In 2021, Chinese guidelines for percutaneous interventional treatment of common congenital heart diseases (7) also recommended the indications for PFO interventional closure. At present, an increasing number of medical centers are beginning to carry out this occlusion, so improving the effectiveness of closure is of great significance.

The Amplatzer PFO occluder is the most widely used occluder in the world at present. It has a self-retractable double disc structure and is sewn into two discs with nickel titanium wire and polyester fabric patches (16). Wahl et al. (17) studied 620 patients who used an Amplatzer PFO occluder for PFO closure, the results showed that all occlusions were successfully completed. At 6 months, transesophageal echocardiography showed that 91% of patients were completely occluded. The probability of preventing recurrent ischemic stroke, TIA or peripheral embolism was 99% in 1 year, 99% in 2 years, and 97% in 5 years, with good safety and clinical efficacy. At present, the commonly used domestic occluders are similar in structure, safety and effectiveness. Liu Wenjuan et al. included 123 PFO patients with CS and large RLS. There was no significant difference in the effectiveness and safety of postoperative follow-up between the Amplatzer PFO occluder group and the domestic cardi-o-fix PFO occluder group (18). Previous studies have confirmed that the complete occlusion rate of patients can reach more than 90% after 12 months of occlusion (17), and the long-term clinical efficacy and safety can be confirmed. However, there is no relevant clinical trial to compare the short-term efficacy of symmetric or asymmetric occluders.

In this study, 92 patients were included. Different types of occluders were selected according to the results of TEE and TTE. There was no significant difference in the ultrasound findings between the two groups, considering that PFO did not affect cardiac structural changes in general, that patients were not randomized but were selected according to the timing of occlusion and then the cardiac structure of patients with PFO was close to normal. During the follow-up period, there were no recurrent strokes, complications of occlusion or death events. At the same time, there was no significant difference in the operation x-ray exposure time and hospitalization time between 45 patients in the symmetrical group and 47 patients in the asymmetric occluder group. During the postoperative follow-up period, there were no new complications, such as atrial fibrillation, pericardial effusion or occluder removal, suggesting that there was no difference in the safety of the two groups. The negative rates of blocking residual shunts in the asymmetric group were higher than those in the symmetric group at 1 month, 3 months, 6 months and 12 months after the occlusion (*χ*^2^ = 6.473, *P* < 0.05; *χ*^2^ = 4.305, *P* < 0.05; *χ*^2^ = 4.842, *P* < 0.05; *χ*^2^ = 4.034, *P* < 0.05). The results showed that the short-term curative effect of the asymmetric group may be better than that of the symmetric group. Compared with the symmetrical occluder, the asymmetric occluder had a relatively smaller left disc. Having the structure close to the PFO makes the device more stable while not only blocking the right to left shunt but also facilitating adhesion and endothelial coverage between the device and the surrounding tissues and creating better adherence to the atrial septum. Although the left disc with a large symmetrical occluder can effectively cover the entire PFO fissure, it failed to closely fit the atrial septum. On the one hand, it will rub against the aorta, which may erode the atrial wall. On the other hand, the normal endothelialization time after occlusion will also be relatively increased. Whether the occluder is endothelialized is closely related to the closure efficacy. Endothelialization of the device can help avoid proliferation of fibrous tissue caused by friction with the atrioventricular wall and also prevent direct contact with the blood flow, reduce adverse stimulation and reaction, make the blood flow more stable, and effectively reduce the possibility of thrombosis (19). Incomplete endothelialization of the occluder is also an important reason for residual shunts after occlusion (20). Residual RLS after transcatheter closure is related to the effect of stroke prevention and migraine relief. Gianluca et al. showed that the existence of a permanent shunt was associated with the highest risk of stroke recurrence (OR value: 5.9, 95% CI: 2.0–12, *P* < 0.001) (21). Deng et al. found that residual shunts was associated with an increased risk of stroke or TIA recurrence (HR value: 3.05, 95% CI: 1.65–5.62, *P* < 0.001). The cumulative probability of stroke or TIA recurrence 5 years after closure was 9.3% in patients with residual shunts and 2.5% in patients without residual shunts (22). Another study (23) also suggested that residual RLS after occlusion can be used as an independent predictor of whether migraine is relieved. Jilin University conducted a study on 217 Chinese people with migraine and found that a close correlation has been documented between RLS and migration (24). Therefore, the selection of an appropriate occluder is helpful for reducing residual shunts after occlusion, thereby preventing the recurrence of stroke and alleviating migraine. It is worth mentioning that the negative rate of residual shunts in both groups increased significantly 12 months after occlusion. Considering that the two types of occluder can eventually achieve complete endothelialization over time, it can also be inferred that there may be no significant difference in the long-term efficacy of the two types of occluder.

During the follow-up, no safety events occurred in either group. There was no significant difference in MIDAS scores between 25 migraine patients in the symmetrical group and 28 migraine patients in the asymmetric occluder group before occlusion (*P* = 0.528). The MIDAS scores of patients in the symmetrical group were significantly higher than those in the asymmetric group at 6 and 12 months after the occlusion (*P* = 0.038; *P* = 0.049), indicating that for patients with PFO and migraine, the short-term efficacy of the asymmetric occluder was better than that of the symmetric occluder.

In conclusion, the asymmetric Amplatzer and its similar PFO occluder have a better short-term closure effect than the symmetric occluder, with less postoperative residual shunts, but there was no significant difference in safety between the two groups. This study also has limitations. Patient grouping was chronological only, randomization was not performed, and baseline levels were not appreciably different, although some selection bias may still have occurred. Due to patient compliance problems, some patients did not complete the 12-month follow-up, and the follow-up method should be strengthened in future studies. The conclusions need to be confirmed by larger prospective clinical studies.

## Data Availability

The raw data supporting the conclusions of this article will be made available by the authors, without undue reservation.
