# Supplementary material for: Comparison of the short-term efficacy of different Amplatzer models and similar occluders in the treatment of patent foramen ovale
Source: Front Cardiovasc Med. 2023 Jul 11;10:1092465. doi: 10.3389/fcvm.2023.1092465 (PMC10366722; doi:10.3389/fcvm.2023.1092465)
Supplement: Supplementary file 1 [file Datasheet1.docx]

**Comparison of the short-term efficacy of different Amplatzer models and similar occluders in the treatment of patent foramen ovale**

Yuxuan Lou^a,b,†^, Yang Hua^a,†^, Jing Shi^a^^,†^, Fengze Yang^a^, Yifei Wang^a^, Yang Yang^a^, Wei Sun^a^, Xiangqing Kong^a,b,*^ and Hao Zhang^a,*^

^a^ Department of Cardiology, The First Affiliated Hospital of Nanjing Medical University, Nanjing, Jiangsu, China; ^b^ Southeast University, Nanjing, Jiangsu, China

* Corresponding authors:

E-mail addresses: [kongxq@njmu.edu.cn](mailto:kongxq@njmu.edu.cn) (Xiangqing Kong), [haozhanghh@163.com](mailto:haozhanghh@163.com) (Hao Zhang). ^†^ These authors contributed equally to our study

Sources of Funding: Jiangsu Province Hospital Clinical Capacity Improvement Project (JSPH-MB-2020-7)

Table.1 Comparison of large RLS after occlusion between symmetric and asymmetric groups

| Item | Symmetry group | Asymmetric group | χ2 | *P value* |
| --- | --- | --- | --- | --- |
| Within 3 days after occlusion | n=45 | n=47 | 5.484 | 0.019 |
| large RLS | 21 | 11 |  |  |
| Non-large RLS | 24 | 36 |  |  |
| 1 month after occlusion | n=42 | n=46 | 5.146 | 0.023 |
| large RLS | 14 | 6 |  |  |
| Non-large RLS | 28 | 40 |  |  |
| 3 months after occlusion | n=37 | n=45 | — | 0.088 |
| large RLS | 3 | 0 |  |  |
| Non-large RLS | 34 | 45 |  |  |
| 6 months after occlusion | n=35 | n=41 | — | 0.209 |
| large RLS | 2 | 0 |  |  |
| Non-large RLS | 33 | 41 |  |  |
| 12 months after occlusion | n=35 | n=39 | — | — |
| large RLS | 0 | 0 |  |  |
| Non-large RLS | 35 | 39 |  |  |

**Figure 1. X-ray images of two patent foramen ovale occluders used in this study (A：symmetric type; B：asymmetric type)**


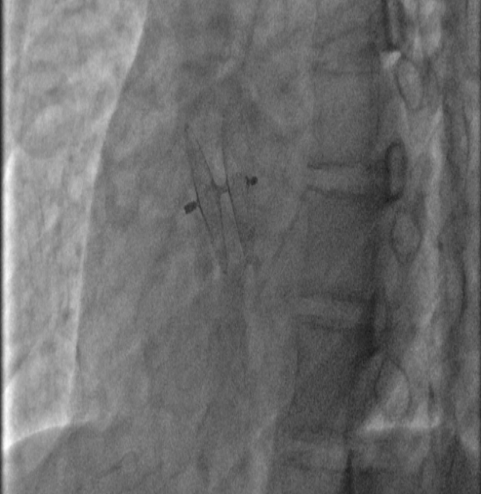

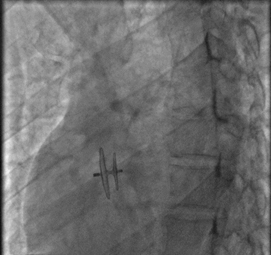


A B
